# Characterization of aldehyde dehydrogenase isozymes in ovarian cancer tissues and sphere cultures

**DOI:** 10.1186/1471-2407-12-329

**Published:** 2012-08-01

**Authors:** Yu-Ting Saw, Junzheng Yang, Shu-Kay Ng, Shubai Liu, Surendra Singh, Margit Singh, William R Welch, Hiroshi Tsuda, Wing-Ping Fong, David Thompson, Vasilis Vasiliou, Ross S Berkowitz, Shu-Wing Ng

**Affiliations:** 1 Department of Obstetrics/Gynecology and Reproductive Biology, Brigham and Women’s Hospital, Boston, MA, 02115, USA; 2 School of Medicine, Griffith Health Institute, Griffith University, Meadowbrook, QLD, 4131, Australia; 3 Department of Pharmaceutical Sciences, University of Colorado Denver, Aurora, CO, 80045, USA; 4 Department of Pathology, Brigham and Women’s Hospital, Boston, MA, 02115, USA; 5 Department of Obstetrics and Gynecology, School of Medicine, Keio University, Tokyo, 160-8582, Japan; 6 School of Life Sciences, The Chinese University of Hong Kong, Hong Kong, China

**Keywords:** Aldehyde dehydrogenase, Isozymes, Ovarian tumors, Sphere cultures, Tumor-type specific expression

## Abstract

**Background:**

Aldehyde dehydrogenases belong to a superfamily of detoxifying enzymes that protect cells from carcinogenic aldehydes. Of the superfamily, ALDH1A1 has gained most attention because current studies have shown that its expression is associated with human cancer stem cells. However, ALDH1A1 is only one of the 19 human ALDH subfamilies currently known. The purpose of the present study was to determine if the expression and activities of other major ALDH isozymes are associated with human ovarian cancer and ovarian cancer sphere cultures.

**Methods:**

Immunohistochemistry was used to delineate ALDH isozyme localization in clinical ovarian tissues. Western Blot analyses were performed on lysates prepared from cancer cell lines and ovarian cancer spheres to confirm the immunohistochemistry findings. Quantitative reverse transcription-polymerase chain reactions were used to measure the mRNA expression levels. The Aldefluor® assay was used to measure ALDH activity in cancer cells from the four tumor subtypes.

**Results:**

Immunohistochemical staining showed significant overexpression of ALDH1A3, ALDH3A2, and ALDH7A1 isozymes in ovarian tumors relative to normal ovarian tissues. The expression and activity of ALDH1A1 is tumor type-dependent, as seen from immunohistochemisty, Western blot analysis, and the Aldefluor® assay. The expression was elevated in the mucinous and endometrioid ovarian epithelial tumors than in serous and clear cell tumors. In some serous and most clear cell tumors, ALDH1A1 expression was found in the stromal fibroblasts. RNA expression of all studied ALDH isozymes also showed higher expression in endometrioid and mucinous tumors than in the serous and clear cell subtypes. The expression of ALDH enzymes showed tumor type-dependent induction in ovarian cancer cells growing as sphere suspensions in serum-free medium.

**Conclusions:**

The results of our study indicate that ALDH enzyme expression and activity may be associated with specific cell types in ovarian tumor tissues and vary according to cell states. Elucidating the function of the ALDH isozymes in lineage differentiation and pathogenesis may have significant implications for ovarian cancer pathophysiology.

## Background

Ovarian cancer accounts for more than half of the deaths due to gynecological malignancy [1]. There were an estimated 14,000 deaths in 2010, thus making it the 5^th^ most common cause of cancer death among women in the United States [2]. As most of the ovarian cancer patients are diagnosed in late stage and 80% of the patients recur despite successful surgery and chemotherapy, the 5-year survival rate is only 30% [3]. Hence, specific and sensitive screening programs and identification of targets that are central to ovarian pathogenesis are of paramount value in decreasing the mortality of ovarian cancer.

Epithelial ovarian cancer is a tumor with great diversity. According to World Health Organization (WHO) criteria, ovarian tumors can be classified as benign, low malignant potential (borderline), or malignant [4]. The histologic classification of ovarian carcinomas is based on morphologic criteria and corresponds to the different types of epithelia in the female reproductive system [5]. There are four major histologic subtypes of epithelial ovarian cancer [4]. Serous tumors are the most common type of ovarian neoplasm with epithelial cells resembling those of fallopian tube and comprise about 50% of primary epithelial ovarian tumors. Mucinous tumors represent 12-15% of epithelial ovarian cancers. They are cystic tumors with locules lined with mucin-secreting epithelial cells resembling either endocervical or colonic epithelium. Recent studies have shown that some mucinous ovarian tumors can be misdiagnosed due to metastasis from other organs [6]. Endometrioid and clear cell tumors each account for 10% of epithelial ovarian cancers. These tumors are thought to arise from foci of endometriosis and endometriotic cysts within the ovary [7,8]. Different tumor subtypes are characterized by dysregulation in specific pathways and have important ramifications in disease prognosis and treatment response [9-11]. Unfortunately, the molecular mechanisms underlying ovarian carcinogenesis and histological differentiation remain elusive.

The cancer stem cell (CSC) model hypothesizes the presence of a cellular hierarchy in the tumors such that a subset of tumor cells have the ability to self-renew and generate the diverse cells that comprise the tumor [12]. CSCs may therefore be responsible for continual sustainment of tumorigenesis, as well as multilineage differentiation into different types of tumors. However, it is difficult to definitively identify cell surface immunophenotypes representing CSCs and their progeny in solid tumors. The cell surface biomarkers described thus far for the same tumor types are found highly variable by different research groups [13-15]. Recently there have been reports showing that differentiated cells can acquire self-renewing capacity [12,16] and stem-like cancer cells arise *de novo* from non-stem cells *in vitro* and *in vivo*[17,18], suggesting bidirectional interconversions between stem and non-stem compartments. Perturbation of the cell-state dynamics by genetic or pharmacological methods has the potential to change the proportions of subpopulations of cells. Hence, it is likely that the “stemness” of a tumor and its response to therapeutic manipulation depends on the stochastic state equilibrium in the populations of cancer cells. The sphere assay discovered in early stem cell studies relies on the capability of stem cells to form spheres when cultured in serum-free medium with growth factors to maintain the undifferentiated state [19]. Mammospheres formed by human mammary epithelial cells exhibit characteristics of early progenitor/stem cells and are able to differentiate along all three mammary epithelial lineages and develop complex functional mammary structures. Tumor sphere cells have recently been widely adopted as an *in vitro* model to study CSCs for human cancers [20-24]. The sphere cells possess self-renewal capacity, with continuous capacity of the dissociated single cells to form secondary spheres. Lower numbers of sphere cells than bulk cancer cells are sufficient to form tumors when transplanted into non-obese diabetic-severe combined immunodeficient (NOD-SCID) mice and show great metastatic capacity [20-24].

Many of the sphere cells and stem cells reported in different systems have been found to be associated with elevated ALDH1A1 enzyme activity as measured by a commercially available kit, Aldefluor® [20,25,26]. Positive correlations between ALDH1A1 enzyme activity and expression are apparent [27], indicating that ALDH1A1 expression or activity may be used with other cell surface markers to identify tumor-initiating cells in hepatocellular, prostate and breast solid carcinomas [28-30]. ALDH1A1 expression has been found to be associated with early metastasis and poor clinical outcome [26]. Aldehyde dehydrogenase (ALDH) proteins are a superfamily of 19 enzymes that are found to protect cells from cytotoxic and carcinogenic aldehydes in various organelles including the nucleus, cytosol, mitochondria, and endoplasmic reticulum [31,32]. The ALDH enzymes also play a crucial role in epithelial homeostasis. Thus, deregulation of these enzymes is linked to multiple cancers, such as breast, prostate, lung and colon cancers [33-37]. In this study, we aimed to investigate if the expression of ALDH isozymes varied among different histological subtypes of ovarian tumor tissues. Our focus was on ALDH class 1, 3 and 7 isozymes, all of which have been reported to be associated with cancer development [28,33-35]. Moreover, as a preliminary approach to explore the potential association between these ALDH isozymes and cancer cells in stem-like state, we have also investigated the expression levels of these ALDH isozymes in ovarian cancer cells growing as spheres in serum-free medium.

## Results and discussion

### Type-specific expression of ALDH isozymes

**Figure 1:**
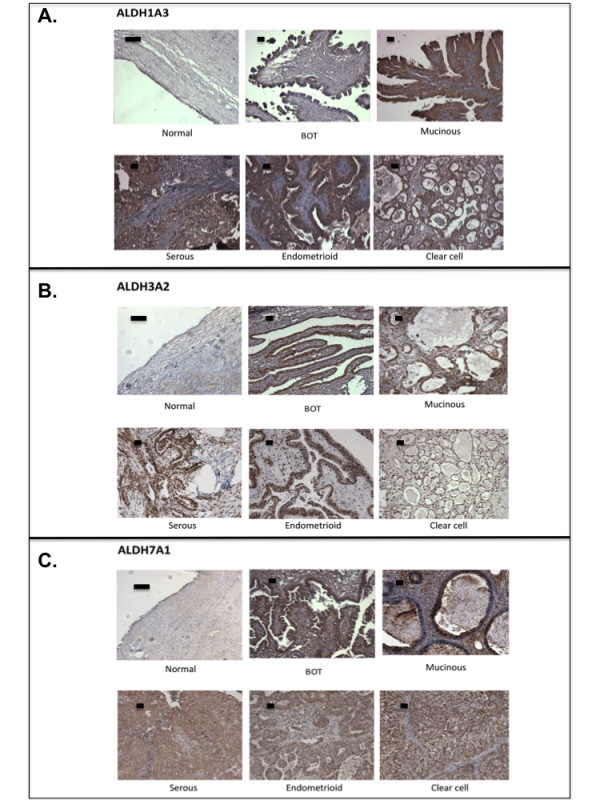
**Expression of ALDH1A3, ALDH3A2, and ALDH7A1 in archived ovarian tissues.** Representative figures of immunohistochemical staining of **A.** ALDH1A3; **B.** ALDH3A2; and **C.** ALDH7A1 in ovarian tissues. BOT, borderline tumors. Scale bar represents 50 μm.

We first employed immunohistochemistry to investigate the expression levels of the different ALDH isozymes in archived ovarian tissues using isozyme-specific antibodies. Antibodies specific to ALDH1A1, ALDH1A3, ALDH3A1, ALDH3A2, ALDH3B1 and ALDH7A1 were used to stain a panel of healthy ovaries, benign, borderline, and invasive ovarian tumors. The clinicopathologic characteristics of the samples we used are shown in Additional file 1: Table S1. We found significantly elevated expression of ALDH1A3 (Table 1), ALDH3A2 (Table 2), and ALDH7A1 (Table 3) in the epithelial ovarian tumor tissues than healthy ovarian epithelia. Multiple comparisons using Dunnett’s method showed that there were significant differences between normal ovaries and invasive tumors for ALDH3A2 and ALDH7A1, whereas ALDH1A3 staining showed significant differences between normal ovaries and both borderline and invasive tumors. There was no significant difference in the staining for ALDH isozymes in normal, benign, and tumor stromal components. There was no positive staining from the ALDH3A1 antibody, and the staining of ALDH3B1 did not show significant differences between healthy ovaries and ovarian tumor tissues (data not shown). Differences between histologic tumor subtypes for ALDH1A3, ALDH3A2, and ALDH7A1 were not significant, partly due to the small and unbalanced sample sizes in these experiments and subtle changes may not be detected. Representative figures of the immunohistochemical staining of ALDH1A3, ALDH3A2, and ALDH7A1 antibodies to different categories of tissues are shown in Figure 1.

**Table 1:** ALDH1A3 immunohistochemical staining according to diagnosis and histological characteristics of the epithelial and stromal components of ovarian samples

***Characteristics***	***n***^**a**^	***epithelial***			***stromal***	
		***score***^**b**^	***P***^**c**^	***C.I.***^**d**^	***score***^**b**^	***P***^**c**^
*Diagnosis*						
healthy	4	0.13 ± 0.25	0.02*	reference	0.13 ± 0.25	0.27
benign	3	3.25 ± 0.43		(-0.8, 7.0)	1.58 ± 1.38	
borderline	3	4.92 ± 2.63		(0.9, 8.7)**	0.75 ± 0.66	
invasive	19	4.28 ± 2.24		(1.3, 7.0)**	0.70 ± 0.98	
*Histology*						
serous	8	3.88 ± 2.84	0.47		0.91 ± 1.31	0.73
mucinous	3	5.00 ± 0.00			0.92 ± 1.16	
endometrioid	4	5.56 ± 1.59			0.25 ± 0.00	
clear cell	4	3.25 ± 2.06			0.56 ± 0.63	

^a^ Number of cases.

^b^ Immunohistochemistry staining score expressed as mean ± SD.

^c^ P-value, ANOVA test of equal means among the four groups (* equal population variances assumption did not meet, corresponding p-value using Kruskal-Wallis test for equal medians was displayed).

^d^ Two-sided 95% confidence interval (C.I.) for the difference in means, Dunnett’s *post-hoc* test compared to value in healthy (in *Diagnosis* group) or clear cell (in *Histology* group) as the “reference” group. Dunnett’s test was performed only when the ANOVA or Kruskal-Wallis result was significant (p < 0.05).

** Significant based on Dunnett’s test (family error rate set to 0.05).

**Table 2:** ALDH3A2 immunohistochemical staining according to diagnosis and histological characteristics of the epithelial and stromal components of ovarian samples

***Characteristics***	***n***^**a**^	***epithelial***			***stromal***	
		***score***^**b**^	***P***^**c**^	***C.I.***^**d**^	***score***^**b**^	***P***^**c**^
*Diagnosis*						
healthy	5	0.94 ± 0.38	0.003*	reference	1.90 ± 0.74	0.1*
benign	4	3.81 ± 1.84		(−1.0, 6.7)	2.25 ± 1.50	
borderline	3	4.42 ± 0.63		(−0.7, 7.7)	1.83 ± 2.02	
invasive	35	5.59 ± 2.60		(1.9, 7.4)**	1.00 ± 0.88	
*Histology*						
serous	17	5.44 ± 2.86	0.31		1.09 ± 0.87	0.74
mucinous	6	5.75 ± 1.17			0.92 ± 1.16	
endometrioid	5	7.50 ± 2.60			0.65 ± 0.78	
clear cell	6	4.54 ± 2.75			1.21 ± 0.87	

^a^ Number of cases.

^b^ Immunohistochemistry staining score expressed as mean ± SD.

^c^ P-value, ANOVA test of equal means among the four groups (* equal population variances assumption did not meet, corresponding p-value using Kruskal-Wallis test for equal medians was displayed).

^d^ Two-sided 95% confidence interval (C.I.) for the difference in means, Dunnett’s *post-hoc* test compared to value in healthy (in *Diagnosis* group) or clear cell (in *Histology* group) as the “reference” group. Dunnett’s test was performed only when the ANOVA or Kruskal-Wallis result was significant (p < 0.05).

** Significant based on Dunnett’s test (family error rate set to 0.05).

**Table 3:** ALDH7A1 immunohistochemical staining according to diagnosis and histological characteristics of the epithelial and stromal components of ovarian samples

***Characteristics***	***n***^**a**^	***epithelial***			***stromal***	
		***score***^**b**^	***P***^**c**^	***C.I.***^**d**^	***score***^**b**^	***P***^**c**^
*Diagnosis*						
healthy	5	0.64 ± 0.47	0.005*	reference	1.70 ± 0.45	0.54
benign	4	2.63 ± 2.29		(-2.2, 6.2)	3.75 ± 0.50	
borderline	3	4.58 ± 1.44		(-0.6, 8.5)	3.00 ± 1.00	
invasive	40	5.36 ± 2.77		(1.8, 7.7)**	3.27 ± 2.67	
*Histology*						
serous	18	4.57 ± 3.09	0.34		2.87 ± 2.53	0.08
mucinous	8	6.44 ± 2.46			5.43 ± 1.86	
endometrioid	8	5.88 ± 2.58			1.70 ± 1.75	
clear cell	5	6.20 ± 1.79			3.25 ± 4.03	

^a^ Number of cases.

^b^ Immunohistochemistry staining score expressed as mean ± SD.

^c^ P-value, ANOVA test of equal means among the four groups (* equal population variances assumption did not meet, corresponding p-value using Kruskal-Wallis test for equal medians was displayed).

^d^ Two-sided 95% confidence interval (C.I.) for the difference in means, Dunnett’s *post-hoc* test compared to value in healthy (in *Diagnosis* group) or clear cell (in *Histology* group) as the “reference” group. Dunnett’s test was performed only when the ANOVA or Kruskal-Wallis result was significant (p < 0.05).

** Significant based on Dunnett’s test (family error rate set to 0.05).

**Figure 2:**
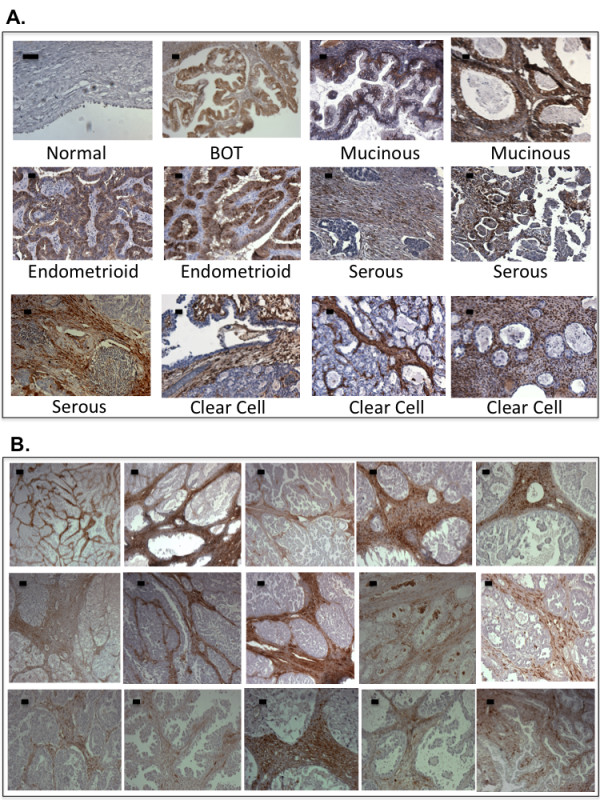
**Expression of ALDH1A1 in archived ovarian tissues. A.** Representative figures of immunohistochemical staining of ALDH1A1 in normal ovaries and different subtypes of ovarian tumor tissues. **B.** Extended panel of fifteen clear cell ovarian tumor samples stained with ALDH1A1 to demonstrate the predominant staining in the stromal fibroblasts. Scale bar represents 50 μm.

Initial immunohistochemical staining of the stem cell marker ALDH1A1 in ovarian tissues yielded particularly interesting patterns not seen with the other ALDH isozymes described above. We have therefore added more cases to confirm the initial findings and the final results are presented here. The staining was not significantly different between healthy ovaries and ovarian tumors (Table 4). However, we saw significant differences in the expression between the different histologic subtypes of ovarian tumors. The endometrioid and mucinous tumors had significant overexpression in the epithelial tumor cells, whereas serous and clear cell epithelial tumor tissues showed very low ALDH1A1 expression (*P* < 0.001). Dunnett’s multiple comparisons showed significantly lower expression of ALDH1A1 in clear cell tumors than in mucinous and endometrioid tumor types. While the epithelial clear cell tumor cells showed lower ALDH1A1 expression than the other tumor types, ALDH1A1 expression was higher in the clear cell stromal fibroblasts than in the other stromal tumor types (*P* = 0.02). Figure 2 shows representative images of ALDH1A1 in the different ovarian tissues and in particular the absence of ALDH1A1 staining in the tumors but increased staining in the stromal part of eighteen clear cell ovarian tumors.

**Table 4:** ALDH1A1 immunohistochemical staining according to diagnosis and histological characteristics of the epithelial and stromal components of ovarian samples

***Characteristics***	***n***^**a**^	***epithelial***			***stromal***		
		***score***^**b**^	***P***^**c**^	***C.I.***^**d**^	***score***^**b**^	***P***^**c**^	**C.I.**^**d**^
*Diagnosis*							
healthy	5	0.15 ± 0.22	0.102		0.60 ± 0.82	0.08	
benign	4	1.94 ± 2.96			4.44 ± 3.14		
borderline	8	3.78 ± 3.37			1.94 ± 1.92		
invasive	101	1.66 ± 2.71			1.85 ± 2.25		
*Histology*							
serous	40	0.67 ± 1.52	< 0.001*	(-0.8, 1.5)	1.12 ± 1.28	0.02	(-3.1, -0.5)**
mucinous	12	5.65 ± 3.05		(3.7, 7.0)**	1.98 ± 2.17		(-2.7, 0.9)
endometrioid	19	3.38 ± 3.28		(1.6, 4.5)**	1.76 ± 2.70		(-2.7, 0.4)
clear cell	29	0.31 ± 0.45		reference	2.90 ± 2.74		reference

^a^ Number of cases.

^b^ Immunohistochemistry staining score expressed as mean ± SD.

^c^ P-value, ANOVA test of equal means among the four groups (* equal population variances assumption did not meet, corresponding p-value using Kruskal-Wallis test for equal medians was displayed).

^d^ Two-sided 95% confidence interval (C.I.) for the difference in means, Dunnett’s *post-hoc* test compared to value in healthy (in *Diagnosis* group) or clear cell (in *Histology* group) as the “reference” group. Dunnett’s test was performed only when the ANOVA or Kruskal-Wallis result was significant (p < 0.05).

** Significant based on Dunnett’s test (family error rate set to 0.05).

**Figure 3:**
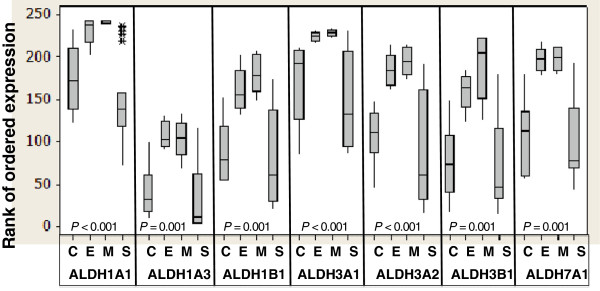
**Boxplot to show the quantitative reverse transcription-polymerase chain reaction results of ALDH isozymes in the ovarian cancer cells present in different subtypes of ovarian tumor tissues.** RNA was extracted from tumor cells microdissected from 19 high-grade serous, 5 mucinous, 6 clear cell, and 5 endometrioid tumor tissues and quantitative reverse transcription-polymerase chain reactions were performed. Each box covers the middle 50% of ranks of ordered expression of the corresponding ALDH isozyme, and the horizontal line within a box marks the median. The lines extending from a box reach to the minimum and maximum data values, except the presence of outliers that are marked with an asterisk. Kruskal-Wallis P-values are presented to indicate whether the median ranks of the ALDH isozymes are significantly different among the four histologic groups. C, clear cell ovarian tumors; E, endometrioid ovarian tumors; M, mucinous ovarian tumors; S, serous ovarian tumors.

To evaluate the tumor type-specific expression of ALDH isozymes, we also performed quantitative reverse transcription-polymerase chain reaction (qRT-PCR) to measure the mRNA expression levels of *ALDH1A1, ALDH1A3, ALDH1B1, ALDH3A1, ALDH3A2, ALDH3B1* and *ALDH7A1* in tumor cells microdissected from a panel of frozen tumor tissues. Boxplot in Figure 3 shows that, in general, the RNA levels of all ALDH isozymes were significantly higher in both endometrioid and mucinous tumors than in clear cell and serous tumors. The RNA expression patterns resemble the protein expression of ALDH1A1, which shows higher expression in the endometrioid and mucinous tumors compared with clear cell and serous tumors. However, as ALDH1A3, ALDH3A2 and ALDH7A1 isozymes did not show particularly significant tumor-type specific protein expression, there might be other post-transcriptional mechanisms that regulate the different ALDH isoenzyme protein levels in the tumor tissues.

**Figure 4:**
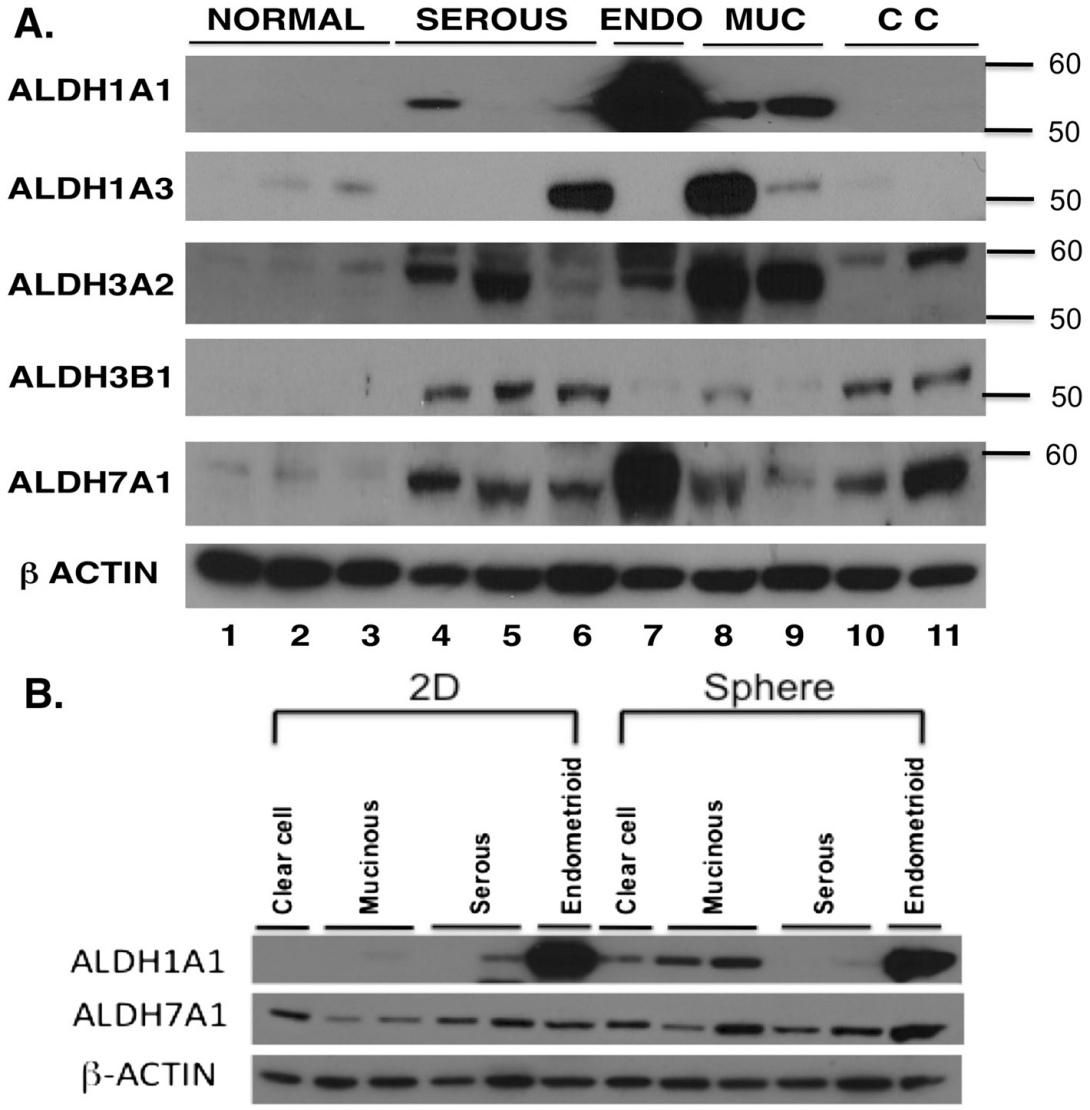
**ALDH isozyme protein expression in ovarian cell lines. A.** Western blot analysis was used to compare the expression of different ALDH isozymes in normal human ovarian surface epithelial (HOSE) cell lines with cancer cell lines of different subtypes, i.e., serous, endometrioid (ENDO), mucinous (MUC) and clear cell (CC). The cell lines were (starting from left): HOSE1-15, HOSE7, HOSE2170, SKOV3, OVCA432, OVCA433, TOV112D, MCAS, RMUGL, RMG1, and OVCA810. Molecular weights are shown on the right. β-actin served as loading control. **B.** Western blot analysis was used to compare the levels of ALDH1A1 and ALDH7A1 in ovarian cancer cell lines growing as a monolayer (2D) or sphere culture. The cell lines were (starting from left): RMG1, MCAS, RMUGL, OVCA432, SKOV3, and TOV112D. β-actin served as loading control.

Western blot analysis was performed to evaluate the expression of the ALDH isozymes in ovarian cell lines. As illustrated in Figure 4A, most of ALDH isozymes except ALDH1A3 showed higher levels in the ovarian cancer cell lines relative to the normal human ovarian surface epithelial (HOSE) cell lines. Only OVCA433 and MCAS showed higher level of ALDH1A3 expression. Like the immunohistochemical staining results in tumor tissues, ALDH1A1 showed a strong tumor type-dependence in expression pattern. While endometrioid and mucinous cancer cell lines showed high protein expression, the serous and clear cell cell lines showed little, if any, detectable protein expression. It is noted that as we have only one endometrioid cancer cell line, the result may not reflect broadly this histologic subtype.

### Expression and activity of ALDH1A1 in ovarian cancer cells growing as sphere suspension

The immunohistochemistry and Western blot results led us to further investigate ALDH1A1 as a potential stem cell marker by evaluating the expression of this protein in ovarian cancer spheres. The sphere assay, which demonstrates the capability of stem-like cells to form spheres when cultured in serum-free medium with growth factors [19], has been widely adopted as an *in vitro* model to study CSCs for human cancers [20-24]. We performed sphere assays with ovarian epithelial cancer cells by growing them as sphere suspensions in standard serum-free medium. We used Western blot analysis to compare the expression of ALDH1A1 and ALDH7A1 in ovarian cancer cells growing as a sphere suspension versus growing as monolayer in complete medium. ALDH7A1 protein expression showed a slight increase in the spheres formed by endometrioid and mucinous cancer cell lines than in the monolayer cells. For ALDH1A1 expression, the two mucinous cancer lines showed increase in expression in sphere cultures than monolayer cultures. The endometrioid cancer cell line expressed very high level of ALDH1A1 both in sphere and monolayer cultures. The clear cell cancer cell line showed some increase from the monolayer cells to the sphere cells. In contrast, serous cancer cell lines did not show any increase in ALDH1A1 expression in the spheres (Figure 4B).

**Figure 5:**
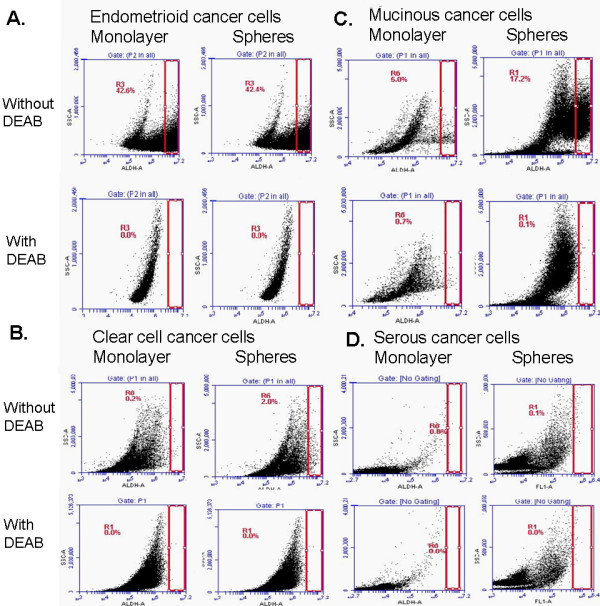
**ALDH activity in cancer cells under different culture growing conditions.** Aldefluor® was used to estimate ALDH enzyme activity in cells grown as a monolayer culture or in a sphere culture. Flow cytometric graphs show the fluorescence intensity of reacted ALDH substrate in the absence and presence of diethylaminobenzaldehyde, a specific ALDH inhibitor, for **A:** endometrioid cancer cell line TOV112D; **B:** Clear cell cancer cell line RMG1; **C.** Mucinous cancer cell line RMUGL, and **D:** high-grade serous cancer cells isolated from clinical ascites. Gated regions indicated ALDH^+^ cells.

In addition to the protein expression analyses, the Aldefluor® assay was used to measure specific ALDH activity in the monolayer and sphere cancer cells. Representative results, shown in Figure 5, are parallel the results obtained by Western blot analysis. The mucinous cancer cells showed robust increased activity in the sphere cells. Endometrioid cells showed strong ALDH activity under both monolayer and sphere conditions. The clear cell cancer cells showed a small increase in activity in the sphere cells, while the serous cancer cells retrieved from an ascites sample did not show any ALDH activity under either culture conditions. Although other studies have suggested that the Aldefluor® assay also measures the activity of some other ALDH isoforms such as ALDH1A3 [34,36], our Aldefluor® assay results closely reflect the ALDH1A1 activity in the cancer cells.

### Implications of ALDH isozyme expression in ovarian cancer

Ovarian cancer is heterogeneous in nature, comprising tumors with different histologic subtypes and developmental stages [4,5]. The cancer stem cell hypothesis proposes the presence of distinct tumor-propagating cell populations that are responsible for self-renewal and multilineage differentiation into different types of tumors [12]. ALDH1A1, and recently ALDH1A3, have been described as valuable stem cell markers in different human tumors and *in vitro* systems [26,28,34,36]. ALDH1A1 positivity has also been associated with chemoresistance in ovarian cancer [38,39]. The present study revealed ALDH1A1 to be expressed predominantly in mucinous and endometrioid epithelial cancer cells, but not in most of the serous and clear cell cancer cells. Instead, high ALDH1A1 expression was found in the stromal fibroblasts in the latter two types of ovarian cancer. In a previous study, higher levels of ALDH1A1 expression were found in mammary stromal cells than in epithelial cells [40]. Although it might be argued that the stromal ALDH1A1 staining arose from cancer cells with mesenchymal features, as suggested in a proteomic profiling study of a panel of lung adenocarcinoma cell lines [37], the predominant stromal staining observed in our study is consistent with ALDH1A1 expression being distinctly lineage-specific in different histologic types of ovarian tumors. It is well documented that expression and activity levels of ALDH isozymes depend on cancer type and/or cell of origin [36,40]. Penumatsa *et al..*. reported recently reduced expression of ALDH1A1 in serous ovarian tumors [41] and Li *et al..*. reported that ALDH1A1 expression was repressed by histone-lysine N-methyltransferase EZH2 in high-grade serous ovarian carcinomas [42]. It will be of great interest to delineate the role of ALDH1A1 in lineage differentiation and its regulation in ovarian cancer.

Moreover, it is of equal importance to evaluate the functional roles of elevated ALDH1A3, ALDH3A2 and ALDH7A1 isozymes in ovarian cancer. The ALDH1A3 isoform has been reported to be a novel CSC marker with potential clinical prognostic application in breast cancer [34]. ALDH7A1 was also found to be involved in prostate cancer bone metastasis [33]. Further analysis of these novel ALDH isozymes may have significant diagnostic and prognostic implications in ovarian cancer. A more thorough understanding of the molecular mechanisms underlying their activities in the development of ovarian cancer may pave a way for more effective treatment of ovarian cancer.

## Conclusions

We have performed an analysis of the expression of different ALDH isozymes in ovarian tumors and cancer cell lines. ALDH1A1 shows a tumor type-specific expression pattern that may indicate a role in lineage-specific differentiation mechanisms during histopathologic development of ovarian tumors. Further studies are required to elucidate the roles ALDH1A1 and other elevated ALDH isozymes play in ovarian pathogenesis.

## Methods

### Ovarian clinical samples and ovarian cell lines

Archived formalin-fixed, paraffin-embedded normal, benign, and cancerous ovarian tissues were collected from women undergoing surgery at the Brigham and Women's Hospital for a diagnosis of primary ovarian cancer or from control subjects who were undergoing the procedure of hysterectomy or oophorectomy for benign gynecologic diseases. Additional 15 cases of clear cell ovarian carcinomas were from Department of Obstetrics and Gynecology, Osaka City General Hospital, Japan. All patient-derived biologic specimens were collected and archived under protocols approved by the Human Subjects Committee of the Brigham and Women's Hospital, USA, and Osaka City General Hospital, Japan. Samples were collected with written informed consent from patients and confirmed histologically by gynecologic pathologists. Cases were staged according to International Federation of Gynecology and Obstetrics (FIGO) system. The normal human ovarian surface epithelial (HOSE) cells and ovarian cancer cell lines have been described previously [43]. Normal HOSE cells were collected by scraping the ovarian surface of the control subjects who were undergoing hysterectomy or oophorectomy for benign diseases. Long-term HOSE cells were immortalized by a HPV E6/E7 gene introduction. All ovarian cell lines were maintained in a mixture of medium 199 and MCDB105 medium (1:1) (Sigma, St. Louis, MO) supplemented with 10% fetal calf serum (Invitrogen, Carlsbad, CA).

### Sphere assay

Standard sphere assay was performed according to Dontu *et al…* with minor changes [19]. Single cancer cells were resuspended in NeuroBasal-A Medium (Invitrogen) supplemented with B27 (Invitrogen), 20 ng/ml EGF and 20 ng/ml bFGF (Invitrogen), and 4 μg/ml heparin (Sigma-Aldrich), in ultra-low attachment culture plates (Fisher Scientific, Pittsburgh, PA). Cells were cultured for 1 week to form spheres before harvesting.

### Immunohistochemistry

Paraffin-embedded ovarian tissue blocks were sectioned at a thickness of 7 μm, mounted on Superfrost Plus microscopic slides (Fisher Scientific, Pittsburgh, PA), and dried at 50 °C for at least 3 hours. Deparaffinization was performed using xylene and rehydration with a graded ethanol series. Antigen retrieval was performed in a pressure-cooker in antigen-unmasking solution (Vector Laboratories, Burlingame, CA) for 10 min. Endogenous peroxidases were blocked using 0.3% H_2_0_2_ in methanol for 20 min. The sections were then blocked with normal blocking serum for 20 min and subsequently incubated overnight with ALDH isozyme-specific antibodies. Antibodies specific to ALDH1A1, ALDH1A3, ALDH3A1, ALDH3A2, and ALDH3B1 have been described [44,45]. Antibody specific to ALDH7A1 was purchased from Epitomics, Inc (Burlingame, CA). After incubations with primary and secondary antibodies (Vector Laboratories, Burlingame, CA), the reaction was visualized using Vectastain Elite ABC Kit with diaminobenzidine chromogen as a substrate (Vector Laboratories, Burlingame, CA). Sections were counterstained lightly with hematoxylin and mounted in Permount® (Fisher Scientific, Pittsburgh, PA). The staining was quantified with a semi-quantitative scoring system. The weighted score was obtained by multiplying the staining intensity score ranging from 3+ (strongest positive) to 0 (no evidence of stain) and the score for the percentage of positive cells ranging from 3+ (100% stained) to 0 (no cells stained). Two trained observers scored the slides independently and the scores were compared for discrepancies and averaged.

### RNA isolation and quantitative reverse transcription-polymerase chain reaction

Microdissection of ovarian tumor cells from frozen tissues (19 high-grade serous, 5 mucinous, 6 clear cell, and 5 endometrioid) was performed using a MD LMD laser microdissecting microscope (Leica Microsystems, Buffalo Grove, IL). Note that these samples were not the same samples used in the immunohistochemical study. Total RNA was isolated using RNeasy Micro Kit (Qiagen, Valencia, CA). Reverse transcription and real-time PCRs were performed using High Capacity cDNA Reverse Transcription Kit and SYBR® Green PCR kit, respectively (Invitrogen Life Technologies,Carlsbad, CA). The primers for different ALDH isozymes are listed in Table 5. To calculate the relative expression for each gene, the 2^−ΔΔCT^ method was used to relate the C_T_ values of ALDH expression in each sample to the C_T_ values for the housekeeping gene cyclophilin A [46].

**Table 5:** Primer sequences used in the quantitative reverse transcription-polymerase chain reactions

***Gene***	***Forward Primer***	***Reverse Primer***
ALDH1A1	5'-ACTGCTCTCCACGTGGCATCTTTA-3'	5'-TGCCAACCTCTGTTGATCCTGTGA-3'
ALDH1A3	5'-ACCTGGAGGTCAAGTTCACCAAGA-3'	5'-ACGTCGGGCTTATCTCCTTCTTCC-3'
ALDH1B1	5'-TGCTGCAGAGTGTCAGCAT-3'	5'-GGTGGTAGGGTTGACCGTCG-3'
ALDH3A1	5'-TGTGTCAAAGGCGCCATGAGCAAG-3'	5'-GGCGTTCCATTCATTCTTGTGCAG-3'
ALDH3A2	5'-TGATTATAAAGCCTTCTGAACTGAGTGAAA-3'	5'-ATGCGTCTGCAAACAATGTCCAGG-3'
ALDH3B1	5'-ACAAGTCAGCCTTCGAGTCGG-3'	5'-AGCACCACACAGTTCCCTGC-3'
ALDH7A1	5'-AGGAGAGGTTTGGGAGAAGTCTGT-3'	5'-TATAAACAGTCGCCTCGCAGTGGT-3'
Cyclophilin A	5'-CTGGACCCAACACAAATGGTT-3'	5'-CATGCCTTCTTTCACTTTGCC-3'

### Western blot analysis

Total cell lysates were prepared from growing cells using RIPA buffer (50 mM Tris–HCl pH 8, 150 mM NaCl, 1% NP-40, 0.5% sodium deoxycholate and 0.1% SDS) supplemented with PhosStop phosphatase inhibitor cocktail and complete protease inhibitor cocktail (Roche Applied Science, Indianapolis, IN) and protein concentration was measured with a MicroBCA protein assay kit (ThermoScientific, Rockford, IL). Ten μg of total cell lysates were resolved by sodium dodecyl sulfate-polyacrylamide gel electrophoresis (SDS-PAGE) and transferred to a polyvinylidene fluoride (PVDF) membrane using a SEMI-DRY Transfer cell (Bio-Rad Laboratories, Hercules, CA). After blocking with 5% nonfat dry milk in 1X TBST buffer (10 mM Tris–HCl pH 7.5, 150 mM NaCl, 0.05% Tween-20) at room temperature for 1 hr, the membrane was incubated with the primary antibody at 4°C overnight, then washed at room temperature with 1X TBST buffer. The bound antibody was detected by the secondary antibodies conjugated with horseradish peroxidase (Pierce Biotechnology, Rockford, IL) and a Supersignal west pico kit (Pierce Biotechnology, Rockford, IL).

### Aldefluor® assay

ALDH activity was detected using the Aldefluor® assay kit (STEMCELL Technologies, Vancouver, BC, Canada) as described by the manufacturer. Briefly, dissociated single cells from cell lines or spheres were resuspended in Aldefluor® assay buffer containing an ALDH substrate, bodipy-aminoacetaldehyde (BAAA), at 7.5 μM, and incubated for 1 hr at 37°C. An identical reaction was also performed in the presence of 15 mM diethylaminobenzaldehyde (DEAB), an ALDH-specific inhibitor. Fluorescence intensity of the stained cells was analyzed using an Accuri C6 Flow Cytometer (BD Accuri Cytometers, Ann Arbor, MI). The reaction with DEAB was used to define the baseline for the assay, i.e., fluorescence not associated with ALDH activity. ALDH activity of a sample was determined based on the fluorescence intensity beyond the threshold defined by the reaction with DEAB.

### Statistical Analysis

All analyses were performed using MINITAB statistical software (Minitab, State College, PA). ANOVA was used to compare the mean IHC scores among different diagnostic and histologic groups. If the equal population variances assumption was not met, the non-parametric Kruskal-Wallis test was performed to compare the results obtained from ANOVA. When there was a statistically significant difference among the groups, multiple comparisons with a control group (Healthy group for Diagnosis and Clear Cell group for Histology) were performed using the Dunnett’s method. A difference was deemed significant when it reached the 5% level, i.e., P ≤ 0.05. As the normality assumption for the qRT-PCR data was not met, the Kruskal-Wallis test was used to analyze the ranked qRT-PCR data according to histologic subtypes, and the results are presented as a boxplot.

## Competing interests

The authors declare that they have no competing interests to disclose.

## Authors’ contributions

Y-TS, MS carried out the immunohistochemistry. JY carried out Western blot analysis, RT-PCR and sphere cultures. SL performed Aldefluor® assays and analysis. SS participated in the RT-PCR. S-KN performed statistical analysis. Y-TS, DT, and S-KN helped draft the manuscript. HT, WRW, VV, and RSB contributed the samples and reagents and critically revised the manuscript. W-PF participated in the design of the study. S-WN conceived, participated in study design and coordination and wrote the manuscript. All authors read and approved the final manuscript.

## Pre-publication history

The pre-publication history for this paper can be accessed here:

http://www.biomedcentral.com/1471-2407/12/329/prepub

## Supplementary Material

Additional file 1 Table S1. Click here for file
